# Modeling of interactions between xenobiotics and cytochrome P450 (CYP) enzymes

**DOI:** 10.3389/fphar.2015.00123

**Published:** 2015-06-12

**Authors:** Hannu Raunio, Mira Kuusisto, Risto O. Juvonen, Olli T. Pentikäinen

**Affiliations:** ^1^School of Pharmacy, Faculty of Health Sciences, University of Eastern FinlandKuopio, Finland; ^2^Computational Bioscience Laboratory, Department of Biological and Environmental Science, Nanoscience Center, University of JyväskyläJyväskylä, Finland

**Keywords:** cytochrome P450, metabolism, xenobiotic, *in silico*, modeling

## Abstract

The adverse effects to humans and environment of only few chemicals are well known. Absorption, distribution, metabolism, and excretion (ADME) are the steps of pharmaco/toxicokinetics that determine the internal dose of chemicals to which the organism is exposed. Of all the xenobiotic-metabolizing enzymes, the cytochrome P450 (CYP) enzymes are the most important due to their abundance and versatility. Reactions catalyzed by CYPs usually turn xenobiotics to harmless and excretable metabolites, but sometimes an innocuous xenobiotic is transformed into a toxic metabolite. Data on ADME and toxicity properties of compounds are increasingly generated using *in vitro* and modeling (*in silico*) tools. Both physics-based and empirical modeling approaches are used. Numerous ligand-based and target-based as well as combined modeling methods have been employed to evaluate determinants of CYP ligand binding as well as predicting sites of metabolism and inhibition characteristics of test molecules. *In silico* prediction of CYP–ligand interactions have made crucial contributions in understanding (1) determinants of CYP ligand binding recognition and affinity; (2) prediction of likely metabolites from substrates; (3) prediction of inhibitors and their inhibition potency. Truly predictive models of toxic outcomes cannot be created without incorporating metabolic characteristics; *in silico* methods help producing such information and filling gaps in experimentally derived data. Currently modeling methods are not mature enough to replace standard *in vitro* and *in vivo* approaches, but they are already used as an important component in risk assessment of drugs and other chemicals.

## Introduction

Modern life is based on the use of chemicals, i.e., substances and their mixtures. The current count of individual substances (compounds) is now approaching 100 million^1^. Therefore the chemical cocktail to which humans and environmental species is exposed contains a great number of different compounds. There is little knowledge on the adverse effects of the vast majority of chemicals. Even drugs (pharmaceuticals) cause sometimes unexpected serious adverse effects despite being subject to extensive non-clinical and clinical studies before reaching the market.

Toxicological risk assessment is mandatory for certain chemicals such as drugs, food additives, pesticides, biocides, industrial chemicals, and the most hazardous natural substances. The manufacture and release of chemicals is strictly regulated in the European Union (EU) by the REACH legislation (Registration, Evaluation, Authorization and restriction of Chemicals). REACH is now the paradigm of a deliberate shift toward a more responsible, sustainable, and green use of chemicals. Companies must today report extensive toxicological information about their chemicals. Novel methods for testing have been developed as a direct response to legislative requirements (Scholz et al., 2013; Nicolotti et al., 2014).

Traditional evaluation of chemicals and drugs for toxicological effects has relied heavily on testing with experimental animals. However, today it is widely acknowledged that to assess all commonly used chemicals, animal testing will not solve the challenge. There is great societal pressure to find alternative testing methods. Computational (*in silico*) models are often cited as methods to reduce animal tests. *In silico* approaches are today widely applied for evaluating multiple aspects of chemical toxicity in man and environment (Cronin and Madden, 2010; Raunio, 2011).

## Role of Metabolism in Biological Effects of Chemicals

To understand the actions, either beneficial or adverse, of substances in the human body, one must know how much of the external dose will reach the sites of action (internal dose), and how soon it will be eliminated from the body. Absorption, distribution, metabolism, and excretion (ADME) are the four steps of pharmacokinetics (or toxicokinetics) that determine the internal dose and the concentration in the target sites of the body. Together metabolism and excretion take care of elimination of xenobiotics, compounds foreign to the body. The common practice of adding the letter T for toxicity in the acronym (ADMET) emphasizes the tight connection between ADME properties and toxic outcomes.

Most living organisms have developed systems to prevent absorption of xenobiotics, to eliminate them and to repair and adapt to damages. The ability of our body to clear xenobiotics involves specific enzymatic pathways developed during evolution to handle natural constituents in the diet. Xenobiotics are subjected to one or multiple enzymatic pathways constituting phase 1 oxidation, reduction and hydrolysis, and phase 2 conjugation reactions. Metabolism usually converts lipophilic compounds into more hydrophilic derivatives that can be easily eliminated from the body, usually via urine. Transporter proteins play an important role in xenobiotic ADME by moving compounds and their metabolites through cell membranes and across different body compartments (Gonzalez et al., 2011).

The phase 1 reactions are mediated by the versatile cytochrome P450 (CYP) enzymes and the more structurally selective flavin-containing monooxygenases (FMO), epoxide hydrolases (EH) and other phase 1 enzymes (other oxidizing, reducing, and hydrolyzing enzymes). The CYP enzymes constitute a large superfamily of heme proteins that metabolize a vast number of exogenous and endogenous compounds. Out of 57 different CYP forms, about 10 hepatic CYPs are responsible for the oxidative metabolism of xenobiotics in humans, and as few as seven CYPs are responsible for metabolism of nearly 90% of all drugs. The CYPs metabolize for example polycyclic aromatic hydrocarbons, aromatic amines, heterocyclic amines, pesticides, and herbicides, and the vast majority of drugs. The most common CYP reaction involves a single oxygen atom insertion from molecular oxygen into an organic molecule in reactions such as hydroxylation, sulfoxidation, epoxidation, *N*-dealkylation, *O*-dealkylation, etc.; hence the name ‘monooxygenase.’ However, the enzyme performs a variety of other transformations, such as desaturation, oxidative dehalogenation, reductive dehalogenation, deformylation, peroxidation, and so on (Pelkonen et al., 2008; Testa et al., 2012; Guengerich and Munro, 2013).

The phase 2 enzymes contain several superfamilies of conjugating enzymes. Among the most important are glutathione *S*-transferases (GST), UDP-glucuronosyltransferases (UGT), sulfotransferases (SULT), *N*-acetyltransferases (NAT), and methyltransferases (MT). These enzymes are mostly involved in inactivation reactions, but may also catalyze formation of toxic metabolites (Gonzalez et al., 2011; Testa et al., 2012). The main metabolizing organ in humans is the liver, with some contribution by the small intestine. Many other tissues contain also xenobiotic metabolizing enzymes. These extrahepatic enzymes usually do not contribute to systemic elimination of drugs, but may produce metabolites with significant local effects (Sevior et al., 2012).

There are two facets to metabolism. First, metabolism leads to termination of the action of a compound and allows excretion of metabolites from the body. Second, the same enzymes sometimes produce metabolites that are reactive and toxic. In these cases parent compounds are often transformed into reactive electrophiles, which react with nucleophilic sites of proteins and DNA and form adducts with them. DNA damage caused by reactive metabolites is the main initial mechanism of chemical carcinogenesis and reactive metabolites also explain a large proportion of the so-called idiosyncratic adverse drug reactions (Park et al., 2011; Bessems et al., 2014). An extensive survey (Testa et al., 2012) of commercial drugs showed that on average each xenobiotic can be converted into six different metabolites. Normally 3% of these retain the original activity of the parent compound, while 7% demonstrate toxic effects. If a single compound is metabolized into 10 or more metabolites, the chances that one of these is toxic are very high.

Information on compound’s ADME properties is critical for risk assessment of chemicals. It is acknowledged that absence of metabolism is a key bottleneck in the development of *in vitro* toxicity tests. External exposure must be translated into internal doses and compared with *in vitro* cell exposure associated with effects (*in vitro*–*in vivo* comparison). Data on ADMET properties of compounds are increasingly generated using *in vitro* and *in silico* tools. Recent advances in molecular modeling of CYPs and other critical proteins demonstrate that it is possible to generate realistic models for them (DeLisle et al., 2011; Pelkonen et al., 2011; Carosati, 2013; Bessems et al., 2014).

In this review we focus on *in silico* methods used for evaluating interactions between xenobiotics and human CYP enzymes. Modeling approaches have been applied also to other phase 1 enzymes, including FMOs (Cruciani et al., 2014) and EHs (Lonsdale et al., 2012) as well as phase 2 conjugating enzymes, including UGTs (Sorich et al., 2008), SULTs (Leyh et al., 2013), and various transporters (Ravna and Sylte, 2012). The important field of *in silico* tools for predicting general ADMET properties is extensively covered in recent reviews (Cronin and Madden, 2010; Pelkonen et al., 2011; Di et al., 2013; Roncaglioni et al., 2013).

## Modeling Methods

**FIGURE 1:**
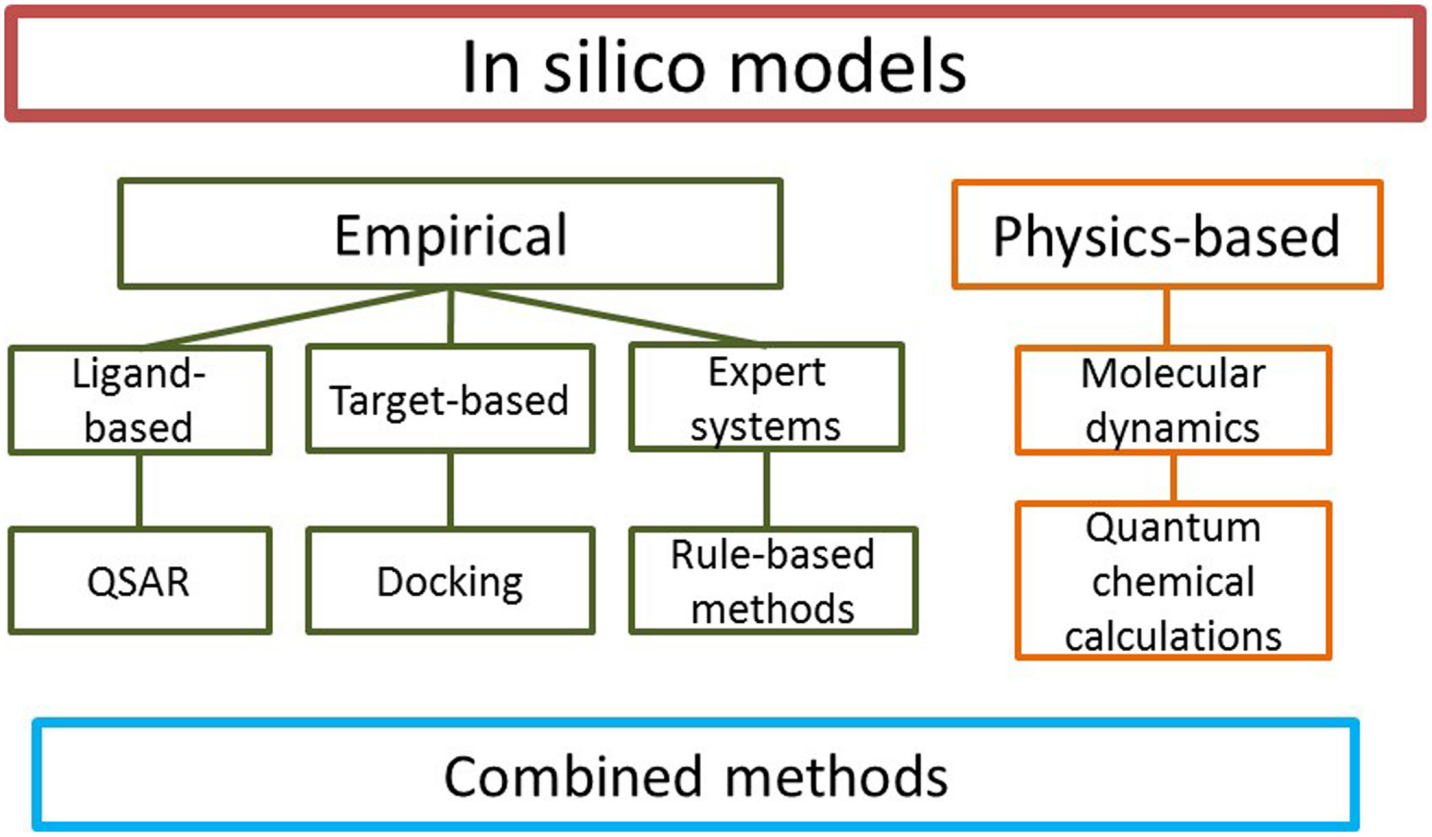
**Types of *in silico* models.** Numerous specific methods exist in each category.

Several different types of *in silico* methods have been developed; the simplest way to classify them is to distinguish physics-based and empirical models (**Figure 1**). Physics-based methods include for example molecular dynamics and the prediction of binding affinity by methods such as free energy perturbation and quantum chemical (QC) calculations. Empirical methods, based on existing experimental data without knowledge of the physics of the system, may be divided to ligand-based and target-based approaches. In ligand-based methods, structures of known active and inactive compounds are modeled to derive quantitative structure-activity relationships (QSARs) and other properties such as sites of metabolism (SOM), i.e., specific atoms in a substrate where metabolic reactions occur. Also various rule-based expert systems belong to this category. In target-based methods, the structure of the enzyme is the starting point for model generation. Models integrating both ligands and enzymes are known as combined or mechanism-based methods.

Quantitative structure-activity relationship methods have evolved from a linear relationships method (Free Wilson method and Hansch analysis) to multiple linear regression methods using grid-based 3-dimensional (3D) QSAR approaches such as Comparative Molecular Field Analysis (CoMFA), Comparative Molecular Similarity Analysis (CoMSIA), and GRID/GOLPE. More statistical intensive methods include neural networks, subsequent variants, and decision trees (Höltje et al., 2003; Sridhar et al., 2012). In CoMFA, ligand-receptor interactions are represented by standard potential energy fields such as steric and electrostatic interactions. Differences in these interaction field intensities in a set of molecules are related to differences in their biological response. Calculation of steric and electrostatic fields is carried out by placing aligned molecules from a dataset into a cubic lattice in which probe atoms surround the molecules. CoMFA uses a partial least-squares (PLS) method in the analysis to predict activity from energy values at the grid points. The results of the PLS analysis are often presented as a 3D coefficient contour map which show favorable and unfavorable steric and electrostatic regions. CoMSIA is based on similarity indices calculated between probe atoms and groups in the same manner as CoMFA fields are calculated. CoMSIA uses a Gaussian function to calculate similarity indices for a data set of pre-aligned molecules at regularly spaced grid points. The similarity indices determine the dependence between the probe atom and the atoms of the molecules in the data set (Höltje et al., 2003).

Expert systems mimic human reasoning and formalize existing knowledge. These are programs in which a computer solves problems by applying rules from a knowledge base. Such rules may be a combination of factual and heuristic types, and are usually non-numerical. In most cases, 3D structures of compounds are not required. Metabolic pathways are sometimes very different even in closely related mammalian species, thus some expert systems allow filtering of specific subsets of the data to a specific species (Kirchmair et al., 2012; Long, 2013). Expert systems exploit the extensive databases of experimentally derived metabolic pathways. Examples of such databases include the Accelrys Metabolite database^2^ and Fujitsu ADME database^3^.

Of the target-based methods, docking analysis mimics the binding of a ligand to a biological macromolecule, usually a protein. Typically, in docking simulation the conformational space of the ligand is sampled within the ligand binding cavity of the target protein to identify the most likely binding conformation(s) for the ligand. The binding affinity or fitness of the ligand is estimated rapidly for all sampled conformations with a scoring function. In principle, docking predicts energetically favorable conformations of ligands and also reveals key groups or atoms for binding. With crystal structures available for the major human CYPs, protein-ligand docking methods are suitable for the analysis and prediction of CYP–ligand interactions. However, docking accounts poorly for substrate reactivity (Kirchmair et al., 2012; Roncaglioni et al., 2013).

Presentation of details of all the *in silico* methods is outside the scope of this review; we will provide here a general view of the state-of-art. Several recent reviews cover the technical aspects extensively (Shaik et al., 2010; Kirchmair et al., 2012; Testa et al., 2012; Cumming et al., 2013; Long, 2013). Today *in silico* methods used to evaluate CYP–ligand interactions typically combine techniques from physics-based and empirical models. With appropriate combinations, the strengths of individual *in silico* methods complement each other.

## Modeling of CYPs

### Selectivity of Ligand Binding

We focus here on the nine most important human liver CYP forms: CYP1A2, CYP2A6, CYP2B6, CYP2C8, CYP2C9, CYP2C19, CYP2D6, CYP2E1, and CYP3A4. The crystal structures of all these CYP enzymes have been elucidated. Ligands to these CYP enzymes are either substrates that are metabolized or inhibitors that decrease substrate turnover. Elucidation of the binding cavities of individual CYPs to their ligand profiles has revealed that the size and shape of the binding cavity are critical for selective ligand binding. A good ligand is able to complement the binding cavity in size, shape, and electrostatic interactions.

Numerous *in silico* models on these nine CYPs have been published since the 1990s using various approaches. The very first studies used simple QSAR analyses of small numbers of molecules, and today highly complex combined methods involving 3D-QSAR are routinely employed. Together, these studies have yielded a fairly detailed picture on the main features of ligand-enzyme interactions. The following text and **Table 1** summarize the main characteristics of these CYPs: typical substrates and inhibitors, common features of the ligands, and main characteristics of the enzyme binding cavities and active sites, i.e., the region of binding cavity that is critical for catalysis.

**Table 1:** Cytochrome P450 (CYP) ligands and their common features.

Form	Substrates	Inhibitors	Common features
1A2	Drugs: caffeine, lidocaine, melatonin, theophylline, tizanidine Other: ethoxyresorufin, polycyclic aromatic hydrocarbons, nitroarenes, heterocyclic aromatic amines/amides	Furafylline, ciprofloxacin, enoxacin, α-naphthoflavone	Small, aromatic/planar, lipophilic, acid or neutral, polyaromatic hydrocarbons
2A6	Drugs: nicotine Other: coumarin,	Methoxsalen, tranylcypromine, pilocarpine, 3-(pyridine-3-yl)-1H-5-yl)methanamine	Diverse, relatively small neutral or basic molecules usually containing one aromatic ring
2B6	Drugs: bupropion, cyclophosphamide, efavirenz Other: *n*-hexanes, monoterpenes	Thio-TEPA, ticlopidine, 2-phenyl-2-(1-piperidinyl)propane, 4-benzylpyridine, 2-phenyl-2-(1-piperdinyl)propane	Medium molecular size, hydrophobic; at least one hydrogen bond acceptor possibly near SOM
2C8	Drugs: paclitaxel, amodiaquine, rosiglitazone, repaglinide Other: fatty acids	Trimethoprim, montelukast, acyl glucuronide of gemfibrozil, acyl glucuronide of clopidogrel	Promiscuous hydrophobicity/hydrophilicity features
2C9	Drugs: *S*-warfarin, tolbutamide, diclofenac, flurbiprofen Other: organic solvents	Sulfaphenazole, fluconazole, amiodarone,	Aromatic, lipophilic/non-polar, acid or neutral; possible secondary binding site
2C19	Drugs: omeprazole, *S*-mephenytoin, lansoprazole, diazepam	Omeprazole, ticlopidine	Aromatic, lipophilic, acidic, neutral or basic molecules with site of oxidation a discrete distance from 2 H-bond acceptor heteroatoms
2D6	Drugs: dextromethorphan, bufuralol, codeine, desipramine, atomoxetine Other: tryptamine, insecticides	Quinidine, terbinafine, paroxetine, fluoxetine, sertraline	Flat, positively charged aryl-alkyl-amines with site of oxidation a discrete distance from a protonated nitrogen
2E1	Drugs: chlorzoxazone Other: ethanol, aniline, *p*-nitrophenol, nitrosamines	Pyridine, disulfiram	Small (mw < 100), neutral, hydrophobic molecules, relatively low logP
3A4	Drugs: midazolam, triazolam, nifedipine, felodipine, atorvastatin, lovastatin, ciclosporin A Other: polycyclic aromatic hydrocarbons, endogenous steroids, bile acids	Itraconazole, ketoconazole, indinavir, ritonavir, saquinavir, diltiazem, erythromycin, clarithromycin, gestodene, CYP3cide, SR-9186, mifepristone, raloxifene	Relatively large, lipophilic, structurally diverse molecules, positively charged or neutral with site of oxidation often nitrogen or allylic positions

Data sources: Rendic, 2002; Lewis and Ito, 2010; DeLisle et al., 2011; Khojasteh et al., 2011; Dong et al., 2012; Sridhar et al., 2012; Johnson and Stout, 2013; Zientek and Youdim, 2015. A comprehensive list of drugs metabolized by CYPs can be found at http://bioinformatics.charite.de/supercyp.

#### CYP1A2

The binding cavity of CYP1A2 [Protein Data Bank (PDB) ID 2HI4] is relatively planar and small, with an estimated volume of 375 Å^3^. This binding cavity fits closely with planar compounds, such as the typical CYP1A2 substrates theophylline, caffeine, and melatonin, and the potent inhibitor α-naphthoflavone (Korhonen et al., 2005; Zhou et al., 2010).

#### CYP2A6

The binding cavity of CYP2A6 (1Z10 + others) is rather compact, with a volume of only 260 Å^3^, which is consistent with the fact that CYP2A6 catalyzes the metabolism of small planar substrates, such as coumarin and nicotine (mw 146 and 162 Da, respectively). Coumarin fits excellently in the narrow binding cavity of CYP2A6. The CYP2A6 active site contains three phenylalanines enabling π–π interactions with aromatic compounds and an asparagine forming hydrogen bonding (Yano et al., 2005; Raunio and Rahnasto-Rilla, 2012).

#### CYP2B6

The substrates of CYP2B6 (3QOA + others) are usually non-planar molecules, neutral or weakly basic, fairly lipophilic with one or two hydrogen-bond acceptors; a good example is the antidepressant drug bupropion (Korhonen et al., 2007; Shah et al., 2011; Turpeinen and Zanger, 2012).

#### CYP2C8

CYP2C8 (1PQ2 + others) oxidizes large substrates, such as taxol (mw 854 Da). The binding cavity of CYP2C8 is rather large with a unique shape; its volume is approximately 1450 Å^3^ (Niwa and Yamazaki, 2012; Xiaoping et al., 2013). There are basic residue(s) in the active site as acidic compounds are oxidized efficiently or inhibit the enzyme, for example the acyl glucuronides of gemfibrozil and clopidogrel (Ogilvie et al., 2006; Tornio et al., 2014).

#### CYP2C9

CYP2C9 (1OG5 + others) metabolizes medium-sized acidic molecules with 1–2 hydrogen bond acceptors. The crystal structure of CYP2C9 shows that Arg108 plays a significant role in the binding of acidic substrates such as flurbiprofen (Mo et al., 2009a,b; Niwa and Yamazaki, 2012).

#### CYP2C19

Typical substrates of CYP2C19 (4GQS) are medium-sized molecules, mostly basic with 2–3 hydrogen bond acceptors. The tertiary structures of 2C19 and CYP2C8 are highly similar, although their binding cavities differ greatly due to amino acid differences that directly alter the topography and the hydrophobic and polar landscapes of the cavities (Niwa and Yamazaki, 2012; Reynald et al., 2012).

#### CYP2D6

CYP2D6 (2F9Q + others) binds substrates containing a basic nitrogen and a planar aromatic ring as its active site contains acidic residues. A crystal structure of CYP2D6, in combination with mutagenesis data, indicates that the negatively charged residues, Asp301 and/or Glu216, are involved in substrate recognition and binding (de Groot et al., 2009; Wang et al., 2009).

#### CYP2E1

The binding cavity of CYP2E1 (3E4E + others) deduced from first two crystal structures for this enzyme is the smallest (190 Å^3^) yet observed for a human CYP. This structural knowledge has helped in understanding why CYP2E1 generally catalyzes small molecular substrates, such as acetaminophen and halothane (Porubsky et al., 2008; Yamazoe et al., 2011; Martikainen et al., 2012).

#### CYP3A4

CYP3A4 (1W0G + others) is a very versatile enzyme capable of oxidizing bulky substrates, such as cyclosporine and erythromycin (mw 1203 and 734 Da, respectively). The substrate-free CYP3A4 crystal structure displays a large substrate-binding cavity with a volume of about 1400 Å^3^ (Zhou, 2008; Sevrioukova and Poulos, 2013).

Numerous *in silico* models have given important insights into the nature of interactions between individual CYP forms and their ligands (substrates and inhibitors). These studies have revealed that the rate of CYP-mediated metabolism is likely to be represented by a combination substrate logP and ionization energy, whereas substrate binding can be described by linear combination of several terms, including logP, number of hydrogen bonds and number of π–π stacking interactions between ligand and enzyme, together with the number of rotatable bonds on the substrate molecule which are restricted on binding to CYP (Lewis and Ito, 2010).

To be of practical use, the main parameters to be predicted for CYP-mediated metabolism are: (1) reactions catalyzed, and SOMs and K_m_ and V_max_ values for the reaction, and (2) inhibition of CYP-specific reactions and the inhibition mechanism and key constants (e.g., k_i_).

### SOM Prediction

Ability to predict and identify metabolites of candidate drug molecules is essential to modern drug discovery, because it is crucial to know if the metabolites are active or inactive or possibly reactive and thus toxic. Unfavorable metabolic pathways may exclude a drug candidate from further development, as they may cause toxicity in later, more costly phases of development. The same information is also critical when elucidating the possible effects of any xenobiotic in the body. Many bioactivation pathways to reactive metabolites are known; therefore specific structural alerts are scrutinized especially in drug candidates (Kalgutkar et al., 2005; Stepan et al., 2011).

Oxidation of substrates by CYPs is a multistep process. The rate-determining step involves hydrogen or electron abstraction from the substrate followed by oxygen rebound or a concerted oxygenation via formation of a complementary interaction between the substrate and amino acid residues in the active site near the oxygen coordinated to heme iron. Thus, hydrogen abstraction energy is an important determinant for SOM of a substrate. However, the most reactive site of a substrate may not be the predicted SOM, because different sizes, shapes, and electrostatic forces of complementary interaction in the active sites of various CYPs determine the orientation of substrate toward to the activated oxygen coordinated to heme. Active site differences make the regioselectivity of oxygenation reactions CYP form-specific. It is thus necessity to consider substrate-enzyme recognition in predicting SOMs (Stjernschantz et al., 2008; Lewis and Ito, 2010; Kirchmair et al., 2012; Cruciani et al., 2013).

Various ligand-based and target-based as well as combined methods have been used for SOM prediction. Examples of these methods are given in **Table 2**. Ligand-based methods concentrate on finding common trends and patterns of size, shape, and atomic or physicochemical environment of the substrates and their relation to SOM. Methods used include pharmacophore and QSAR models, fragment analysis and atomic environment fingerprints, often in combination with rule systems. Target-based methods focus on discovering active conformations for substrates in the CYP active site. Target-based SOM prediction utilizes docking, homology modeling, molecular dynamics simulations and fingerprints.

**Table 2:** Examples of *in silico* programs for SOM prediction.

Program/reference	Description	Homepage
**Target-based methods**		
Tarcsay et al. (2010)	SOM selection is based on docking and binding energies of substrates’ metabolites.	–
Vasanthanathan et al. (2009)	Active conformations of CYP1A2 substrates are recognized by docking and binding energy calculation.	–
**Ligand-based methods**		
META-PC	Predicts the structure of likely metabolites; uses a genetic algorithm to prioritize a large biotransformations dictionary; uses also QC descriptors.	multicase.com/meta-pc
MetabolExpert	Predicts the structures of likely metabolites using a database containing rules including substrate and metabolite listings; also contain lists of substructures which inhibit or promote the reaction	compudrug.com/metabolexpert
Meteor Nexus	Knowledge-based software; integrated to SMARTCyp.	lhasalimited.org/products/meteor-nexus.htm
MetaPrint2D (Boyer and Zamora, 2002; Boyer et al., 2007; Adams, 2010)	A data-mining tool that identifies SOMs based on circular fingerprints and fragment-based substrate-metabolite occurrence ratios.	www-metaprint2d.ch.cam.ac.uk/
RS-WebPredictor (Zaretzki et al., 2011, 2012)	Generates pathway-independent, CYP form-specific regioselectivity. Models built with machine learning techniques using numerous QC and topological descriptors.	reccr.chem.rpi.edu/Software/RS-WebPredictor/
SMARTCyp (Rydberg et al., 2010, 2013; Rydberg and Olsen, 2011, 2012)	SOM prediction tool that utilizes fragment-based reactivity and accessibility factors.	farma.ku.dk/smartcyp/
XenoSite (Zaretzki et al., 2013)	Uses both atomic and molecular descriptors in CYP form-specific models built with machine learning methods.	http://swami.wustl.edu/xenosite/
Tyzack et al., 2014	Form-specific machine learning models that use only 2D topological fingerprints as descriptors.	–
**Combined methods**		
MetaSite (Zamora et al., 2003; Cruciani et al., 2005, 2013, 2014)	Identifies likely SOMs by considering reactivity and complementarity of substrate and CYP catalytic site 3D fingerprints; not training set dependent.	moldiscovery.com/software/metasite/
Tyzack et al. (2013)	Utilizes tethered docking, QC activation energies and molecular dynamics.	–
DR-Predictor (Huang et al., 2013)	Combines docking-derived binding energies to atomic descriptors in CYP form-specific models built with machine learning methods.	–
StarDrop P450	Uses AM1 hydrogen atom transfer energy calculations combined with accessibility descriptors.	optibrium.com/stardrop/stardrop-p450-models.php
IMPACTS (Campagna-Slater et al., 2012)	Combination of docking, transition state modeling, and rule-based substrate reactivity prediction.	http://molecularforecaster.com/products.html fitted.ca/impacts.html

Two basic assumptions are made when docking is used to find active conformations for substrates. First, the SOM or at least one of multiple SOMs should be at close proximity to the heme iron. Second, the binding energy of the substrate should be low. These basics are taken into account when the success of the method is validated. Further requirements are that there are no other atoms between the heme iron and the target oxidized atom. Docking is always CYP form-specific, since it relies on the 3D structure of the binding cavity of the particular enzyme. However, as docking predicts only the active conformation of a substrate, performing it alone is not enough to predict specific SOMs.

The flexibility of CYP enzymes needs to be taken into account in modeling. However, the fastest and most basic way is to use rigid CYP crystal structures, which often leave little space of freedom for active conformations. The physical space of rigid structures is often specific for the cocrystallized ligand due to the induced fit effect, and this may mask the true active conformation of the enzyme for another substrate. Induced fit effects have been considered by docking substrates to multiple structures crystallized with varying ligands, flexible structures, or ensembles of a CYP enzyme from molecular dynamics (Hritz et al., 2008; Danielson et al., 2011; Kingsley et al., 2014). Ensemble docking is a time-consuming process and thus it is rational to use only a few target protein structures. Although computationally expensive, molecular dynamics on enzyme-substrate complexes is also a valuable tool for confirming active conformations and flexibility of CYP binding cavities (Park and Harris, 2003).

A common approach to identify a successful docking pose is to require that a substrate’s SOM is within a specific distance from the heme iron in a conformation of lowest binding energy. Often the maximum distance is 6 Å (Hritz et al., 2008; Vasanthanathan et al., 2009). A long radius from the iron leads to a wide accepted area above the heme plane in many CYP structures, leaving space for other substrate atoms besides a SOM. Having multiple substrate atoms in the accepted space leaves the method very error-prone if one wants to define the primary SOM at atomic precision. One approach to overcome this inaccuracy is tethered docking, which forces a specific atom close to the iron, sampling all possible SOMs in a substrate (Tyzack et al., 2013).

Docking is a valuable tool for predicting active conformations for CYP substrates and form-specific regioselectivity. The major drawbacks of docking result from insufficient scoring functions, the flexibility of CYP enzymes, and the inability to predict SOMs precisely. Hydrophobic interactions are important binding forces in many CYP enzymes, but many binding energy algorithms take them poorly into account. The complex CYP reaction cycle involving reactions of oxygen in the active site also makes the estimation of favorable substrate binding more complex. Inaccuracies of docking methods have been compensated by combining docking with different ligand-based methods. Many of these offer atom-specific information. This is a valuable addition to docking, since the individual properties of substrate atoms can then be considered together with favorable conformations of the whole substrate in the active site. Combined methods account for both steric and physicochemical hindrances of the protein and common patterns and reactivities of the substrates and their substructures. Pharmacophore and QSAR models have been used for docking and rescoring predocked substrates to confirm active conformations. SOM prediction is often more accurate if the activation energies of different metabolic reactions are considered in scoring functions (de Groot et al., 1999a,b, 2002; Park and Harris, 2003; Campagna-Slater et al., 2012; Kingsley et al., 2014). Docking and scoring of all possible phase 1 metabolic products of CYP substrates also gives valuable information on regioselectivity (Tarcsay et al., 2010).

Besides docking, target-based SOM prediction can be based on comparison of the active site and substrate fingerprints. Mono-dimensional histogram fingerprints, called correlograms, are generated for all substrate atoms and the heme iron in the CYP active site (Boyer and Zamora, 2002; Zamora et al., 2003; Cruciani et al., 2005). A single correlogram describes distances from an atom to hydrophobic, hydrogen bond donor, and hydrogen bond acceptor atoms or surface areas, respectively, for substrates and the active site. The atomic fingerprint of a substrate that gives the best match for the active site is predicted as the top SOM. Fragment-based reactivity has also been applied to the scoring function, and 3D fingerprints have proven to be even more accurate in this approach (Cruciani et al., 2005, 2013).

Chemical reactivity is an important factor in CYP regioselectivity. Hence, many methods have used activation energies for SOM prediction. The basic principle in estimating the reactivity of different atomic regions in a CYP substrate is to calculate the energy differences between the substrate and all possible reaction intermediates. The traditional approach is to calculate energies for each molecule as a whole (Korzekwa et al., 1990; Jones et al., 2002). Since these QC calculations are slow to perform and have to be done for each substrate separately, more recent methods use fragment-based approaches for calculating activation energies (Singh et al., 2003; Rydberg et al., 2010). The reactivities are precalculated for small molecular fragments, such as an aromatic ring or a methyl group. Substrate substructures can be matched to fragments and assigned activation energies based on the matching fragment.

Both whole-molecule QC calculations and fragment-based reactivities correlate well with experimental activation energies. However, considering reactivity alone is not sufficient for SOM prediction. This is true especially for CYP forms that have fairly constrained binding cavities with orienting amino acid side chains. Substrates of these CYPs require more consideration of the enzyme-substrate interactions when SOM predictions are made. Since the orientation of an atom to the heme iron is an important factor for CYP regioselectivity, solvent accessible surface areas of substrate and relative distances from the center of the molecule have yielded useful additions to SOM prediction methods that are based on reactivity (Singh et al., 2003; Rydberg et al., 2010, 2013). Substrates for certain CYP forms have common functional groups that interact with particular amino acid residues in the active site; thus, these residues position the substrate into a certain binding conformation in the active site. For these substrates, the intramolecular distance of each potential SOM to the functional group can be taken into account in form-specific scoring functions (Rydberg and Olsen, 2011, 2012).

In addition to reactivities, fragments, and accessibility factors, SOMs can be depicted with a vast amount of other topological and QC atomic descriptors. Descriptors can be used to build rules of different complexities for SOM prediction. In human-built expert systems, different descriptors are examined by an expert to build rules of typical SOM characteristics. Manual gathering of rules can be demanding and time-consuming and complex descriptor dependencies can be difficult to build. Data mining methods utilize automated statistical analysis more extensively, while machine learning is a fully automated method for building complex rules. An example of a data mining method uses statistics to find occurrence ratios of substrate fragments and their metabolites from a vast substrate-metabolite database (Boyer and Zamora, 2002; Boyer et al., 2007; Adams, 2010). These ratios are used when SOMs are predicted for novel compounds, the assumption being that the fragment of substrate that corresponds to the highest substrate-metabolite occurrence ratio has the highest chance of being metabolized. Recently, many machine learning methods have been used for creating more complex rules for SOM prediction. The complexity of descriptor sets in these approaches vary significantly from a few to several hundred descriptors (Sheridan et al., 2007; Zheng et al., 2009; Hasegawa et al., 2010; Zaretzki et al., 2011, 2012; Kirchmair et al., 2013). Most descriptors are atom-specific and illustrate the topological and QC properties of an atom and its close environment, such as reactivity, charge and solvent accessibility. Some investigators have reported combinations of atomic and molecular descriptors, including binding energies from docking as well as the flexibility, solubility, and volume of substrate (Huang et al., 2013; Zaretzki et al., 2013). The probably most simple descriptor sets include only atomic environment fingerprints (Rudik et al., 2014; Tyzack et al., 2014).

The main drawback of expert, data mining and machine learning systems is that they require training sets or previous knowledge of substrates and their SOMs. To achieve comprehensive rules for a wide array of metabolic reactions, the training set should be big and diverse, which is not always possible. Also, the training set should be free of errors, especially inaccurate structures (chemical composition, ionization, chirality, etc.). To decrease the presence of errors and inconsistencies in the training set, compounds must clear a chemical curation workflow. There are several ones published in the literature and they ensure the correctness of compounds prior to QSAR analysis. When the rules for SOM prediction are based on restricted chemical space, the models might not be sufficient to cover rules for novel compounds that do not have corresponding structures in the training set. Thus, it is crucial to have large databases to train the models. On the other hand, CYP form or reaction specific models are relatively fast to generate, provided that there is sufficient knowledge about the respective substrates and their SOMs. As could be expected, CYP form-specific models are most useful since the most important descriptors vary between different forms (Sheridan et al., 2007; Huang et al., 2013).

A shortcoming of most current methods of SOM prediction is their inability to predict the relative abundance of the various metabolites. The latest version of the popular MetaSite program (MetaSite 4) deals with this basic problem (Cruciani et al., 2013). Another novel method, developed in the framework of the Human Cytochrome P450 Consortium Initiative^4^, is based on automatic structure elucidation (MassMetaSite). This method is capable of predicting both phase 1 and phase 2 reactions in biomatrices; it also reads experimental data to compare predictions with experiments to automatically elucidate structures, rate of formation, pathways, phenotyping, and kinetic analysis (Zamora et al., 2013).

### Prediction of CYP Inhibition

Inhibition of CYPs can lead to unwanted drug–drug interactions due to the resulting large variations of drug concentrations between patients at target and off-target sites. Within drug discovery, CYP inhibition can cause delays in the progression of candidate drugs and premature closure of projects. Therefore inhibition potency and mechanism need to be predicted early in drug development. The European Medicines Agency (EMA) and the US Food and Drug Administration (FDA) have issued guidance about drug interaction studies. These outline recommendations about a range of studies to evaluate drug–drug interaction potential. Although the most recent versions of these guidances suggest ways to assess potential interactions mediated by phase 2 enzymes and transporter proteins, the main focus is still on CYP enzymes (Prueksaritanont et al., 2013).

One common way to evaluate metabolizing CYPs is *in vitro* incubation of test compounds with microsomes isolated from human liver. By including CYP form-selective inhibitors in the reaction, one can assign the observed metabolism to a particular CYP form. Although there are very potent and selective inhibitors for some CYP forms, for example sulfaphenazole for CYP2C9, the standard inhibitors for some other CYPs need to be improved especially regarding CYP selectivity (Pelkonen et al., 2008, 2011). Inhibiting a specific CYP reaction with a series of structurally related compounds is a fast and economical strategy to obtain SAR information about the CYP binding cavity. However, it has turned out to be more challenging to build *in silico* models for prediction of CYP inhibition than SOMs. There are several reasons for this. (1) As the binding cavities of CYPs can be large and flexible, inhibitor molecules can coordinate directly to heme, bind close to heme or at a distant site in the protein. (2) Several ligands may bind simultaneously. (3) The inhibitor may be oxidized to an electrophilic reactive intermediate, which forms covalent bonding with the CYP protein causing mechanism-based inhibition. The most notable cases of drug–drug interactions have resulted from a perpetrator drug causing such mechanism-based irreversible inhibition of a CYP enzyme mediating the metabolic clearance of a target drug (Pelkonen et al., 2008; Orr et al., 2012).

A recent review by Sridhar et al. (2012) provides a comprehensive account of QSAR studies on CYP–inhibitor interactions. Studies on the major human CYPs 1A1, 1A2, 1B1, 2A6, 2B6, 2C9, 2C19, 2D6, 2E1, 3A4, and some other CYP forms are detailed in the review. These QSAR analyses have provided important insights into the nature of the compounds that can act as inhibitors of the individual CYP enzymes. However, QSAR models are unable to deal with properties involving multiple inhibitory mechanisms and non-linear correlations. Attempts have been made recently to develop general CYP inhibitor docking protocols with enough accuracy and speed to be used in the drug discovery setting. In a recent paper Brändén et al. (2014) show cases where structural information on complexes with CYP2C9/CYP3A4 and inhibitors have been successfully applied in drug discovery projects. By solving the CYP structure crystallized with a test compound, key features of the CYP-inhibitor interactions can be deduced that are not evident from QSAR or general understanding of CYP binding.

There are numerous common substructures mediating mechanism-based inhibition of CYPs (Fontana et al., 2005). Their orientation toward heme is crucial for inhibition. The importance of the non-productive binding mode leading to mechanism-based CYP inactivation is discussed recently by Kamel and Harriman (2013). They illustrate the utility of *in silico* approaches to address bioactivation with emphasis on general mechanistic aspects of mechanism-based inactivation. A recently published technology provided predictions of CYP inhibition, metabolic stability and form selectivity (Carosati, 2013). Three hundred compounds were evaluated *in vitro* for CYP inhibition, metabolic stability, and form selectivity using CYP2C9, CYP2D6, and CYP3A4. Different orientations of a compound within the binding cavity were used to define productive binding modes which differentiate metabolite production and non-productive binding modes which imply that the compound occupies the catalytic site without reacting, causing reversible and often potent CYP inhibition.

**FIGURE 2:**
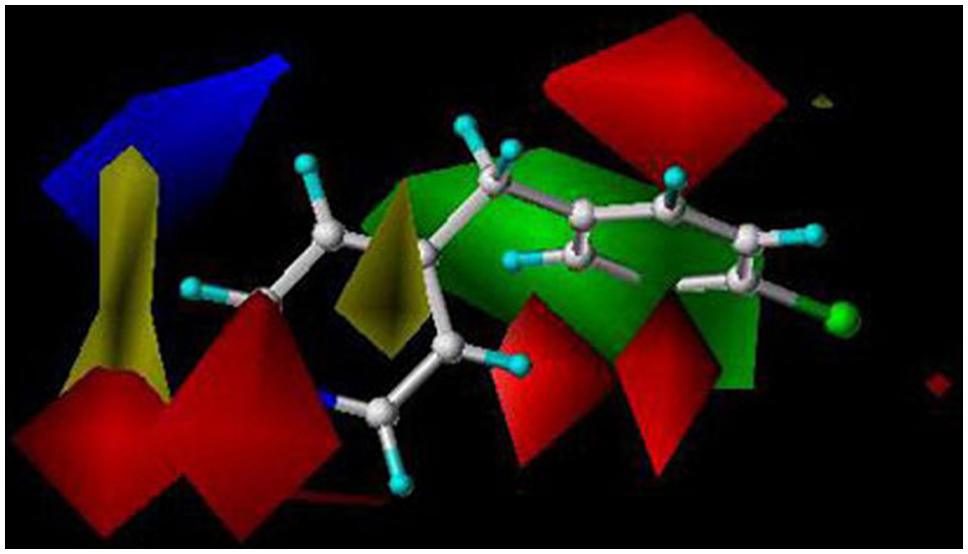
**A CYP2B6 CoMFA model created with 41 training set and seven test set compounds.** The predictive power of the model was very good (*r*^2^ = 0.85). Red and green in the color contour map represent areas where more negative partial charge and bulkier groups increase inhibition potency, respectively. Blue and yellow represent areas where more negative partial charge and bulkier groups decrease inhibition potency, respectively. The reference structure is 4-(4-chlorobenzyl)pyridine. Reproduced with permission from John Wiley & Sons (Korhonen et al., 2007).

**FIGURE 3:**
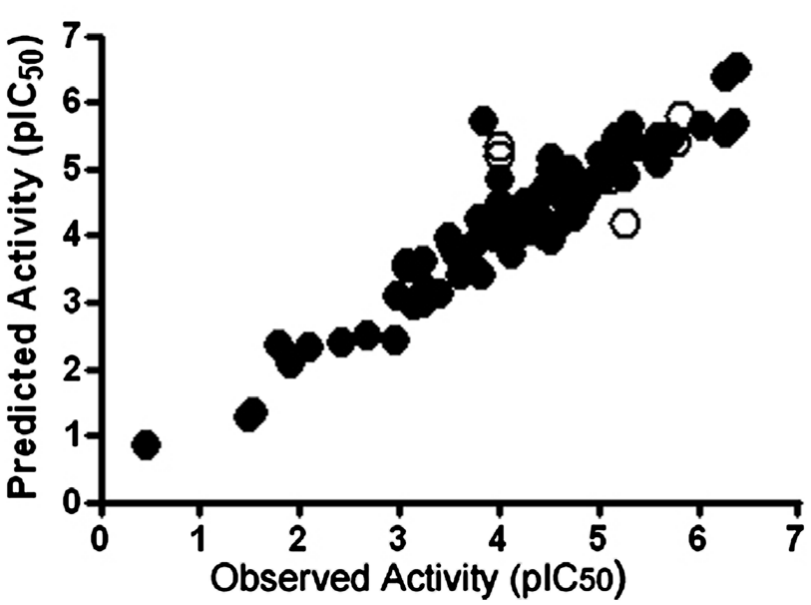
**Plots of observed versus predicted CYP2A6 inhibitory activities of the test (closed circle, *n* = 85) and training (open circle, *n* = 10) set compounds in a CoMFA model.** The outlier molecules differed structurally from the test set molecules. pIC50 value refers to inhibition potency. Reproduced with permission from Elsevier (Rahnasto et al., 2011).

We have used the CoMFA method to evaluate the key molecular interactions between inhibitory compounds and several human liver CYPs, including CYP1A2 (Korhonen et al., 2005), CYP2A6 (Rahnasto et al., 2008, 2011; Tani et al., 2014), CYP2B6 (Korhonen et al., 2007), and CYP2E1 (Martikainen et al., 2012). Several predictive CoMFA models created for CYP2B6 facilitated the discovery of novel potent and selective CYP2B6 inhibitor molecules [4-(4-chlorobenzyl)pyridine and 4-(4-nitrobenzyl)pyridine; Korhonen et al., 2007]. **Figure 2** illustrates key electrostatic and steric properties in the CYP2B6 CoMFA model with 4-(4-chlorobenzyl)pyridine as the model compound. These compounds were later used by others to elucidate the CYP2B6 crystal structure (Shah et al., 2011). We have also identified several novel inhibitors of CYP2A6 using CoMFA/CoMSIA and docking methods together with virtual screening of compound databases (Rahnasto et al., 2008, 2011; Tani et al., 2014). Our experience is that when constructed for relatively small series of structurally related molecules and involving specific individual CYPs, 3D-QSAR models are able to predict inhibitory potencies of unknown compounds with good accuracy. As an example, a CoMFA model for CYP2A6 predicted quite accurately the inhibition potency of compounds in an external test set, even though some of the test compounds were structurally different from those in the training set (**Figure 3**; Rahnasto et al., 2011).

## Metabolism as Part of ADMET Prediction Models

From the critical role played by metabolism in the effects and toxicity of drugs and other chemicals it is clear that any prediction of compound outcomes must take into account its metabolic pathways. Most *in silico* methods still focus on CYPs, but it is obvious that CYP-mediated metabolism is only one component in clearance, which itself is just one aspect contributing to overall effects of xenobiotics.

Multiple *in vitro* methods are today successfully used to generate various ADMET parameters, and this data is increasingly being integrated into models of whole body pharmacokinetics. Prediction of metabolic clearance is an example of a mature *in vitro*–*in vivo* extrapolation (IVIVE) area. Clearance prediction, and IVIVE in general, is increasingly combined with physiologically based pharmacokinetic (PBPK) models to allow prediction not limited to specific parameters but to generate a time course of compound pharmaco/toxicokinetics. PBPK models are built either ‘bottom–up’ using *in vitro* data on ADME or ‘top–down’ based on observed *in vivo* pharmacokinetic parameters. The utility of *in vitro* data to provide quantitative prediction of real-life pharmacokinetic behavior is well established. PBPK models have been used in environmental toxicology and are becoming a vital component of modern drug development (Rowland et al., 2011; Rostami-Hodjegan, 2012; Houston, 2013).

Data from *in silico* approaches are being increasingly used as input to PBPK models. However, although considerable advances have been made, there is still a general lack of quantitative correlation of *in silico* data to *in vivo* ADME parameters. For example, prediction of accurate *V*_max_ and *K*_m_ values (and thus intrinsic clearance *V*_max_/*K*_m_) and hepatic or renal clearance parameters in the human body is still impossible using only data from *in silico* models. The knowledge obtained in *in silico* metabolism of drugs has not yet been transferred to predict the rate of metabolism in PBPK models of toxicological interest because of the differences in the key CYPs involved in metabolism and physicochemical properties of target compounds. Consequently, predictability of hepatic clearance of toxic compounds is limited, mostly focusing of a chemical class or closely related chemicals (Pelkonen et al., 2009, 2011; Coecke et al., 2013; Bessems et al., 2014).

In environmental toxicology, a number of attempts have been made to develop quantitative property-property relationships (QPPRs) based on steric/hydrophobic characteristics, molecular connectivity indices, QC calculations, and so on. These QPPRs have either focused on modeling *V*_max_ or *K*_m_ and not both parameters for a given set of chemicals. Furthermore, these approaches appear to show the promise in modeling single enzyme substrates or a specific reaction and thus are of limited predictive ability (Peyret and Krishnan, 2011).

Several efforts have been made to integrate *in silico* data of CYP-mediated metabolism into prediction of toxic endpoints. One such approach is the VirtualToxLab (Vedani et al., 2014), a system which evaluates the toxic potential of chemicals with endpoints such as endocrine and metabolic disruption, and some aspects of carcinogenicity and cardiotoxicity. The technology involves an automated protocol that simulates and quantifies the binding of small molecules to a series of 16 proteins, known or suspected to trigger adverse effects: 10 nuclear receptors, four CYP enzymes (1A2, 2C9, 2D6, 3A4), a cytosolic transcription factor (aryl hydrocarbon receptor) and a potassium ion channel (hERG). The toxic potential of a compound is derived from its computed binding affinities to these proteins. Thus, this particular technology takes into account metabolism by some key CYPs.

The OECD QSAR Toolbox is a public program for identification of relevant structural characteristics and potential mechanism or mode of toxic action of a chemical. A crucial feature of the Toolbox is grouping chemicals into chemical categories, allowing the ‘read-across’ of information from one chemical to another. Notably, a search is made of the Toolbox databases for known liver or skin metabolites. If these are not found they can be predicted using the metabolism simulators within the program (Sullivan et al., 2014).

A recent paper by Toshimoto et al. (2014) describes a model to predict the major clearance pathways of drugs based on their basic physicochemical properties and descriptors for selected CYP-mediated metabolism, transporters, and renal excretion. These types of studies will take us closer to being able to predict clearance of test compounds based on *in silico* data without need for *in vitro*/*in vivo* input.

## Conclusion

We briefly reviewed the various *in silico* approaches used for predicting outcomes of CYP-mediated metabolism and how this information is integrated into estimation of ADMET endpoints. Evaluation of therapeutic or toxic effects of a xenobiotic should always take into account its ADME properties as those define the internal dose at the site of action. Only after quantitatively combining all ADME aspects one might be able to predict a real-life therapeutic or toxic endpoint. CYP enzymes are key players among the many enzyme and transporter systems affecting compound’s ADMET properties.

*In silico* prediction of CYP-ligand interactions have made crucial contributions in understanding

(1) determinants of CYP ligand binding recognition and affinity
(2) prediction of likely metabolites from substrates
(3) prediction of inhibitors and their inhibition potency

The advantages of *in silico* approaches in assessment of ADMET parameters are clear: they offer very high throughput with reasonable cost. Ligand-based, target-based, and combined methods have yielded very precise information about key features in ligands and binding cavities of all major human xenobiotic-metabolizing CYP enzymes. In drug development, metabolic stability and SOMs of lead molecules can be predicted *in silico* very early in the process. Accurate prediction of SOMs is helpful in optimizing lead molecules for metabolic stability and identifying putative toxic metabolites. However, *in silico* SOM prediction methods cannot yet replace traditional experimental assays, but there is no doubt that they can provide significant insight; hence they are widely used in drug discovery projects within the pharmaceutical industry. Other *in silico* approaches are able to predict drug–drug interaction liabilities due to inhibition of CYP enzyme activity.

Although a lot of progress has been made to address metabolic activation of xenobiotics, a challenge is still presented by the lack of complete understanding of the biological mechanisms of cellular toxicity following exposure to some, but not all, chemically reactive metabolites. Models that predict compound bioavailability and metabolism in particular are now recognized as key components in integrative chemical risk assessment. As greater understanding is obtained about the physiological processes involved, the more reliable predictions will become. PBPK models are being increasingly used for evaluation of compound pharmaco/toxicokinetics. The most advanced PBPK models use *in vitro* data as input, but attempts are made to integrate also *in silico* data. It is clear that in the future there will be an expansion of the ability to link PBPK with improved *in silico* tools to predict ADMET properties.

The various *in silico* procedures for investigating CYP-mediated metabolism can be viewed as complementing those from experimental investigations and also representing a means of interpreting findings from laboratory based studies. The constant development of new algorithms will empower *in silico* analysis methods further leading to the availability of more information about features affecting CYP enzyme-ligand interactions. It is widely believed, however, that computational approaches will not fully substitute *in vitro* and *in vivo* methods in the foreseeable future. Further improvement and regulatory acceptance of data obtained by *in silico* methods will inevitably be determined by experimental confirmation and feedback.

In conclusion, there is a great need to develop and use reliable *in silico* models to predict the bioprofile of chemicals. Information about CYP-mediated metabolism of chemicals is just one piece in a bigger puzzle when making predictions about toxic outcomes in humans or environmental species. This is, however, a crucial piece of information without which reliable predictive *in silico* methods cannot be developed.

## Conflict of Interest Statement

The authors declare that the research was conducted in the absence of any commercial or financial relationships that could be construed as a potential conflict of interest.

## Footnotes

1 www.cas.org

2 http://accelrys.com/products/databases/bioactivity/metabolite.html

3 www.fujitsu.com/jp/group/kyushu/en/solutions/industry/lifescience/admedatabase

4 www.moldiscovery.com/consortia/CYP
